# Fat Intake and Stress Modify Sleep Duration Effects on Abdominal Obesity

**DOI:** 10.3390/nu11102535

**Published:** 2019-10-21

**Authors:** Sangwon Chung, Chang Keun Kwock

**Affiliations:** Korea Food Research Institute, 245, Nongsaengmyeong-ro, Iseo-myeon, Wanju-gun, Jeollabuk-do 55365, Korea

**Keywords:** sleep duration, abdominal obesity, fat intake, stress, effect modification

## Abstract

Though the association between sleep duration and obesity has been generally acknowledged, there is little information about the mechanisms behind this association. The purpose of this study was to examine the effect of the fat intake and stress variables on the association between sleep duration and abdominal obesity. Data for 13,686 subjects aged ≥ 20 years from the 2013–2017 Korea National Health and Nutrition Examination Survey were used, and hierarchical and stratified logistic regression analyses were employed. In the hierarchical logistic regression analyses, fat intake and stress did not change the significance or the size of the sleep effects upon abdominal obesity. These results suggest that sleep duration does not affect abdominal obesity through fat intake or stress variables. In addition, fat intake and stress are not mediators of the sleep duration variable. However, subjects with different levels of fat intake and stress showed different associations between sleep duration and abdominal obesity. Subjects who were in the lowest or highest group of fat intake as well as self-reported stress level showed a weaker relationship between sleep duration and abdominal obesity, compared with the other groups. In conclusion, fat intake and stress modify the effects of sleep duration on abdominal obesity according to the stratified regression results.

## 1. Introduction

Obesity is an unresolved global issue, despite long-term research efforts and active government interventions. Accumulated fat in abdominal adipose tissue has been reported to be closely associated with hyperlipidemia, insulin resistance and inflammation [1]. Abdominal obesity which is diagnosed as an increased waist circumference or waist-to-hip ratio has been recently suggested to be a better predictor of the risk of chronic diseases than obesity [2,3,4].

Conventional obesity research has approached obesity problems through the aspect of the energy balance between calorie intake and expenditure. However, research interests have started to extend toward psychiatric factors, such as short sleep duration, stress and depression. While a minimum of 7 h (420 min) of sleep is recommended for adults [5], the time spent sleeping is decreasing. In Korea, the average time spent sleeping was 460 min (7 h, 40 min) in 2009, according to the Organization for Economic Cooperation and Development (OECD) statistics, but it was reduced to 395 min (6 h, 35 min) in 2013 [6]. With an ever-increasing population with a short sleep duration (SSD), obesity and sleep duration have drawn the attention of obesity researchers from many disciplines of science. Overall, a meta-analysis has found a positive association between SSD and the incidence of obesity or increased waist circumference, and other diseases or mortality among adults [7,8,9,10,11,12,13]. Among Asian adults, less than 6 h (<360 min) and more than 9 h (>540 min) of sleep duration have a 31% and 64% higher risk of obesity, respectively [14]. 

An association of sleep behaviors, including bedtime and the difference between the shortest and longest sleep duration in a week, with obesity, has also been examined. A large difference of sleep duration and late bed time are positively associated with obesity [15]. Similarly, evidence acknowledging the association between sleep duration and obesity is accumulating, but research efforts to understand the nature of this association are rare.

Two mechanisms relating SSD and obesity may be physiologically explained as follows: (1) SSD increases ghrelin, an appetite-stimulating hormone, and decreases leptin, an appetite-suppressing hormone, in plasma [16,17,18]; (2) SSD also increases the secretion of cortisol [19], which results in increased calorie intake and the total consumption of foods [20]. This aspect of association between sleep duration and hormones affecting appetite and food intake can be understood through energy metabolic processes. There is abundant evidence that sleep duration affects dietary factors, such as total calorie intake, high intake of fatty foods, high intake of carbohydrates, and frequent snacking [21,22,23,24]. Therefore, short sleep results in a higher total calorie intake, thereby increasing the risk of obesity [25,26,27]. There are also studies suggesting that a high fat diet can affect sleep duration [28,29]. These two directional studies complete the association between fat intake and sleep. However, other studies have refuted the above causal relationship. Dietary factors cannot fully explain the association between sleep duration and obesity [30,31]. That is, these studies acknowledge the association between sleep duration and obesity, but dietary factors only partially explain the effect of sleep upon body mass index or weight increase.

Short sleep and stress are known as bidirectionally-related variables [32,33,34,35]. Chronic stress increases the secretion of cortisol to cope with stress responses. Stress stimulates the hypothalamic-pituitary-adrenal (HPA) axis and increases the secretion of cortisol, thereby increasing the consumption of comfort, energy dense and/or fatty foods, which promote obesity [36]. Another role of increased cortisol which facilitates visceral fat deposition has been reported by a study using a high-fat/high-sugar diet-fed mice model [37]. Therefore, these effects of stress on abdominal obesity are important.

These physiological studies have suggested that SSD is associated with an increased risk of abdominal obesity, and that fat intake and stress levels are associated with both sleep duration and abdominal obesity. Therefore, the fat intake and stress factors are potential confounding variables, which may obscure the association between sleep duration and abdominal obesity. The purpose of this present study was to examine the role of fat intake and stress in the association between sleep duration and abdominal obesity. Various logistic regression analyses, including hierarchical logistic regression analyses, were employed to examine the effect of the fat intake level and stress variables on the association between sleep duration and abdominal obesity.

## 2. Materials and Methods

### 2.1. Study Population

We used data from the 2013–2017 Korea National Health and Nutrition Examination Survey (KNHANES), which is a representative national survey of Korea for the analysis of this study. KNHANES is ongoing, and has been annually conducted by the Korea Centers for Disease Control and Prevention (KCDC) since 1998. KNHANES consists of a health and life style interview, health examination and nutrition survey. Of the 30,553 adults aged ≥20 years participating in the 2013–2017 KNHANES, we only included 13,686 participants based on the following exclusion criteria: Energy intake < 500 kcal/day or > 5000 kcal/day (*n* = 4128); pregnant or lactating (*n* = 274); taking medicine or undergoing treatment for diabetes, cardiovascular diseases, or thyroid disease (*n* = 9519); or they had missing values on the sleep duration question, their waist circumference and socio-demographic or life-style related variables (*n* = 2946). The survey protocol was approved by the Institutional Review Board of the KCDC, and informed consent was collected from all participants.

### 2.2. Outcome Variables

Abdominal obesity was used as an outcome variable, and subjects were diagnosed as having abdominal obesity if their waist circumference was ≥ 90 cm for men, and ≥ 85 cm for women, according to the criteria standardized for Koreans [38]. The waist circumference of each participant was horizontally measured at the center of the uppermost iliac crest and the lowermost rib cage at the mid-axillary line to the nearest 0.1 cm by trained staff during the health examination.

### 2.3. Independent Variable

Data on sleep duration were obtained from the health interview and used as an independent variable. The participants answered the question, “How many hours a day do you usually sleep?” during the weekdays or weekends, and the average was calculated. The average sleep duration time was rounded to the first digit, and these results were categorized into the following five groups: ≤5, 6, 7, 8 and ≥9 h.

### 2.4. Other Covariates

Covariates included sex, age, smoking, drinking, physical activity, family history of chronic disease, stress level, household income, living area, education and nutrient intake, and these variables were obtained from the health and life style interview or nutrition survey conducted by trained interviewers. Sociodemographic variables included sex, age, household income, living area and education. Living area was categorized into urban and rural, and household income was categorized into ≤middle-upper income and highest income. Education level was divided into ≤middle school graduate, high school graduate and ≥college graduate.

Smoking, drinking, physical activity, family history of chronic disease and stress level were included in the health and life style variables. The participants’ answers to the question, “Do you currently smoke?”, included ‘everyday’, ‘sometimes, ‘smoked in the past but do not currently smoke’ or ‘never’, and they were categorized as follows: ‘everyday’ or ‘sometimes’ were categorized into ‘yes’; and ‘smoked in the past but do not currently smoke’ or ‘never’ were categorized into ‘no’. Drinking data were obtained from the question, “How often did you drink during the year before the interview?”, and participants were categorized into as ‘never or rarely’, ‘2–4/month’, and ‘≥2/week’.

Physical activity was expressed as metabolic equivalent of task (MET) hours/day based on the physical activity questions. The participants answered the question, “How many days, hours, and minutes have you usually spent more than 10 min of physical activity in a week?”, according to the following physical activity levels: Vigorous, moderate, and walking. The number of days and duration time were estimated as hours a day.

Family history of chronic disease was categorized into ‘yes’ and ‘no’ based on the question, “Have your family members ever been diagnosed with chronic disease, such as hypertension, hyperlipidemia, cardiovascular diseases, or diabetes, etc. from a doctor?”. The participants’ answers to the question, “How often do you feel stress in your daily life?”, included ‘rarely stressed’, ‘slightly stressed’, ‘moderately stressed’, or ‘highly stressed’. Finally, calorie, carbohydrate, protein and fat intakes were obtained from the 24-h dietary recall data of the nutrition survey conducted by well-trained dietitians, and the proportions of calorie intake from carbohydrates, proteins and fats were estimated. KNHANES conducts its food intake survey using a 24-h dietary recall method. All food intakes are recorded by energy and nutrition analysis software, and intakes of calories and all the macro- and micro-nutrients are calculated by the software using a nutrition composition database. The proportion of calorie intake from carbohydrate, protein and fat was calculated by percentage of energy derived from each nutrient composition of all food intakes out of their total energy intake. We did not control other dietary variables, such as snacking, because snacks generally are high in fat and/or carbohydrate, and it can be covered by the fat and carbohydrate intake variables.

### 2.5. Statistical Methods

Because the primary objective of this study was to examine the effect of fat intake and stress on the association between sleep duration and abdominal obesity, our initial approach was to set up a logistic regression model as follows: Prevalence of abdominal obesity for dependent variable; sleep duration variables for independent variable; and self-reported stress level variables and quartiles of proportion of calorie intake from fat for the third control variables.

The first test investigated if fat intake and stress variables have a mediating effect. The tests were performed by adding variables sequentially using hierarchical logistic regression analyses [39]. If the fat intake and stress variables are mediators, then the addition of confounder in the logistic regression would mediate the significance and/or size of impact of the sleep variable. The quartiles of the proportion of calorie intake from fat and the self-reported stress level were added on top of the baseline and baseline plus dietary variable models. The hierarchical logistic regression modeling approach was employed to examine the effect of fat intake and stress on the association between sleep and abdominal obesity by sequentially adding control variables. Our baseline model, model 1, consisted of sleep duration variables, namely sex, age, health and lifestyle variables (such as smoking, drinking, physical activity and family history of chronic disease), and other socio-demographic variables (such as household income, living area and education). Model 2 included model 1 variables and the following dietary factor variables: Total calorie intake and the proportion of calorie intake from carbohydrates. Model 3 included model 2 variables plus the quartiles of the proportion of calorie intake from fat or self-reported stress levels.

The second test investigated if the fat intake and stress variables modify the effects of sleep duration on abdominal obesity. To assess the effect modifications by fat intake and stress variables, the effect of sleep duration on abdominal obesity was assessed in stratified groups of fat intake and stress levels [40]. We stratified logistic regression analyses over the quartiles of the proportion of calorie intake from fat and self-reported stress levels, and we examined the sleep duration effects across the stratified logistic regression analyses. To test if the stratified model is statistically superior to the aggregated model, log-likelihood tests were conducted for these logit regression models [41]. This log-likelihood test determines the better model between stratified models and one aggregated data model. If structural breaks across stratified data exist, the log-likelihood test favors the stratified model over the aggregated model.

All statistical analyses were conducted using STATA S/E 15.0 (StataCorp, College Station, TX, USA). For categorical variables and continuous variables, the Chi-square test and one-way analysis of variance (ANOVA) were performed, respectively, to estimate the differences and statistical significance in the distribution across sleep duration. To estimate the association between sleep duration and abdominal obesity, odds ratios (ORs), 95% confidence intervals (95% CIs) and *p*-trends were calculated using multivariable logistic regression analysis with the group of subjects sleeping less than 5 h a day as the reference group. Statistical significance was determined at a *p*-value < 0.05.

## 3. Results

### 3.1. General Characteristics of Study Population

Table 1 shows the general characteristics of the study population according to sleep duration. The average sleep duration of the study population was 7.02 ± 1.3 h/day. The mean age decreased from 51.2 ± 15.8 years in the group of subjects sleeping less than 5 h a day to 42.7 ± 16.5 years in the group of subjects sleeping more than 9 h a day. Subjects sleeping more than 9 h a day tended to be less physically active and had a higher proportion of calorie intake from fat than those sleeping less than 5 h a day. Moreover, subjects sleeping 7 h a day tended to drink less frequently, more frequently had a family history of chronic disease, felt stressed less frequently, had higher household incomes, had higher education levels, and were more likely to live in an urban area.

### 3.2. Effects of Fat Intake on the Association between Sleep Duration and Abdominal Obesity

Table 2 shows the effect of sleep duration on the prevalence of abdominal obesity. The upper panel of Table 2 shows the results of the hierarchical logistic regression analyses with sequentially increasing control variables. Model 1 evaluated the sleep duration effect while adjusting for sex, age, health and lifestyle variables, such as smoking, drinking, physical activity, family history of chronic disease, household income, living area and education. Model 2 adjusted for model 1 control variables plus dietary variables, and model 3 adjusted for model 2 control variables plus the fat intake variable. The group of subjects sleeping more than 9 h a day had a reduced risk of abdominal obesity compared to those sleeping less than 5 h a day with the adjustment of socio-demographic variables in model 1. This negative trend remained after further adjustment for nutrient intake. After adjustment of calorie intake and proportion of calorie intake from carbohydrate in model 2, the highest sleep duration group had a 35% lower risk of abdominal obesity (OR: 0.65, 95% CIs: 0.54–0.78, *p*-trend < 0.001) compared to the lowest sleep duration group. The size of the impact of sleep duration on abdominal obesity remained the same with the further adjustment of the proportion of calorie intake from fat, and the *p*-trend was still significant (*p*-trend < 0.001) in model 3. Because the significance and size of impact of sleep duration on abdominal obesity did not change after the addition of the fat intake variable, the fat intake variable was not a mediator.

The lower panel of Table 2 demonstrates the effects of fat intake levels on the association between sleep duration and abdominal obesity by stratifying the proportion of calorie intake from fat into quartiles. The range and median value of each quartile were as follows: 0.2–13.4% and 9.8% in the lowest quartile, respectively; 13.4–19.4% and 16.4% in the second quartile, respectively; 19.4–25.8% and 22.4% in the third quartile, respectively; and 25.8–61.9% and 30.8% in the highest quartile, respectively. The manner in which sleep duration affected the prevalence of abdominal obesity was different at different levels of fat intake in model 2 compared with the aggregated model. The sleep duration effects on abdominal obesity were attenuated at the highest (fourth) and lowest (first quartile) fat intake groups. In the second and third quartile, the group of subjects sleeping more than 9 h a day was also associated with a 50% (OR: 0.50, 95% CIs: 0.34–0.73, *p*-trend = 0.001) and 41% (OR: 0.59, 95% CIs: 0.40–0.87, *p*-trend = 0.001) lower risk of abdominal obesity compared to those sleeping less than 5 h a day, respectively. The other sleep duration groups were significantly associated with abdominal obesity in the second quartile. Further, the group of subjects sleeping 8 h a day in the third quartile group was negatively associated with abdominal obesity. However, only the group of subjects sleeping more than 9 h a day had a 32% reduced risk of abdominal obesity compared to those sleeping less than 5 h a day (OR: 0.68, 95% CIs: 0.49–0.94, *p*-trend = 0.007) in the lowest quartile. Sleep duration was not associated with abdominal obesity in the highest quartile group. The stratified models were shown to be better than the aggregated model based on the log-likelihood test (*x*^2^ (60) = 118, *p* < 0.01).

### 3.3. Effects of Stress on the Association between Sleep Duration and Abdominal Obesity

The effect of stress on the relationship between sleep duration and abdominal obesity is shown in Table 3. We evaluated the effect of stress using multivariable logistic regression models 1, 2 and 3. The results showed a negative trend, and the group of subjects sleeping more than 9 h a day was associated with a 34% lower risk (OR: 0.66, 95% CIs: 0.55–0.79, *p*-trend < 0.001) of abdominal obesity compared to those sleeping less than 5 h a day with the adjustment of socio-demographic variables, calorie intake, proportion of calorie intake from carbohydrate, and self-reported stress level in model 3. Similar to the result of fat intake, the significance and size of the impact of sleep duration on abdominal obesity were not changed with any further adjustment of self-reported stress level. Thus, stress level was not considered as a mediator.

Next we examined the association between sleep duration and abdominal obesity across the self-reported stress level to confirm if stress modifies the effect. The effect of sleep duration on abdominal obesity had different associations at stress levels in model 2 compared with the aggregated model. No significant association was observed in the lowest and highest stress levels (*p*-trend = 0.063 in the rarely stressed group; *p*-trend = 0.606 in the highly stressed group). However, the highest sleep duration group had a 35% (OR: 0.65, 95% CIs: 0.50–0.83, *p*-trend < 0.001) and 49% (OR: 0.51, 95% CIs: 0.35–0.74, *p*-trend = 0.002) lower risk of abdominal obesity in the slightly stressed and moderately stressed group, respectively. In the log-likelihood test, the stratified models were shown to be better than the aggregated model (*x*^2^(60) = 99, *p* < 0.01).

## 4. Discussion

Evidence for the association between sleep duration and obesity has been accumulating. However, the background information regarding this association is lacking. We examined how potential confounding variables, such as fat intake and stress, affect the association between sleep duration and abdominal obesity. Our findings were as follows: (1) Adding fat intake or stress variables in logistic regression did not influence the significance of sleep duration variables; (2) stratified models including the fat intake level and stress level were superior to the aggregated model, and the associations between abdominal obesity and sleep duration were changed across stratified models according to fat intake and stress levels.

The first finding of this study showed that fat intake and self-reported stress levels are not mediators of sleep duration variables based on the fact that the addition of the fat intake and self-reported stress level variables did not change the significance and/or the size of sleep effects on abdominal obesity risk. This result indicated that the sleep effects on abdominal obesity risk are not explained by fat intake or stress levels [42]. Because our sleep effect was a direct effect that did not act through mediators, the direct effects of sleep duration variables may be nondietary effects, such as a circadian effect, which was not examined in the present study [28]. Our third control variable, fat intake and stress variables, was conceptually in the causal path between sleep duration and abdominal obesity, as previous biophysiological studies have suggested that a short sleep duration increases stress and high-fat food consumption through hormonal changes, thereby increasing the risk of abdominal obesity. However, the results of the present study agree with other studies by Patel et al. [30] and Nishiura et al. [31], suggesting that dietary behaviors, such as calorie and high-fat/high-carbohydrate intake, do not explain much about the association between sleep duration and obesity.

The second finding of this study reported that subjects with different fat intakes showed different associations between sleep duration and abdominal obesity. Subjects with the lowest fat intake (first quartile) or highest fat intake (fourth quartile) showed a weaker association between sleep duration and abdominal obesity compared with subjects with a slightly lower fat intake (second quartile) or slightly higher fat intake (third quartile). Thus, the effect of sleep duration on abdominal obesity was different in subjects with different fat intakes. The models stratified by stress levels also showed modified effects, indicating that the sleep effects on abdominal obesity risk were heterogeneous according to fat intake and stress levels [39]. Both fat intake and stress levels modified the association between sleep duration and abdominal obesity risk [39], this is possibly because there is not much abdominal obesity risk left to be explained by sleep duration for subjects in the lowest and highest quartiles of fat intake and stress. A cautious interpretation of our results is advised, since there could be other variables, like sex, which could modify the effect of sleep on abdominal obesity, even though we found that only fat intake and stress have moderation effects.

The present study contributes to the existing literature by providing evidence that fat intake and stress have moderation effects on the association between sleep and abdominal obesity risk. However, there are some potential limitations in this study. First, the results of the present study were from cross-sectional data analyses. Thus, it was impossible to confirm the causal relationships, but the findings presented in the present study were statistically robust. 

Another potential limitation is that our dietary behavior data were collected by a 1-day 24-h recall food intake survey; therefore, the dietary behavior data contained some errors in measurement, which attenuated the effect on the risk of abdominal obesity. However, this database was proven to be useful and accurate, because the sleep duration effects on abdominal obesity found in our study were similar to those of previous studies, acknowledging the association between sleep duration and obesity. Further research is needed to determine whether the results from this study will hold up in prospective study settings.

## 5. Conclusions

In conclusion, a negative relationship between sleep and abdominal obesity risk was evident; especially, a minimum of 7 h of sleep lowered the risk of abdominal obesity among subjects with healthy fat intake and among slightly or moderately stressed groups. In the aspect of nutritional implication, a healthy amount of fat intake is recommended for the beneficial effect of a minimum of 7 h sleep, which is equivalent to the National Sleep Foundation guideline.

## Figures and Tables

**Table 1:** General characteristics ^a^ of the study subjects according to sleep duration.

	Sleep Duration (Hours/Day)					*p*-Value ^b^
	≤5	6	7	8	≥9	
*n* = 13,686	1355	2983	4189	3523	1636	
Gender						<0.001
Men (%)	38.7	43.1	42.6	41.2	35.3	
Women (%)	61.3	56.9	57.4	58.8	64.7	
Age (years)	51.2 ± 15.8	45.8 ± 14.0	44.6 ± 13.9	43.7 ± 14.5	42.7 ± 16.5	<0.001
Current smoker (%)						0.056
Yes	21.8	19.3	18.1	18.7	18.7	
No	78.2	80.7	81.9	81.3	81.3	
Drinking (%)						<0.001
Never or rarely	57.6	51.9	53.1	52.7	54.9	
2–4/month	19.4	26.4	26.3	25.2	23.4	
≥2/week	23.0	21.8	20.6	22.1	21.8	
Physical activity (MET, hours/day)	4.5 ± 7.7	4.2 ± 6.0	3.8 ± 5.4	3.6 ± 5.2	3.3 ± 5.4	<0.001
Family history of chronic disease (%)	54.8	57.2	60.7	58.6	52.9	<0.001
Stress level (%)						<0.001
Rarely	14.2	12.7	13.8	14.3	16.9	
Slightly	50.7	59.4	61.9	60.9	55.6	
Moderately	26.1	23.6	20.9	21.3	22.4	
Highly	8.9	4.2	3.4	3.6	5.0	
Household income (%)						<0.001
≤Middle-upper income	73.3	66.3	64.0	66.3	72.5	
Highest income	26.7	33.7	36.1	33.7	27.5	
Living area						<0.001
Urban	80.7	83.3	85.2	83.3	78.6	
Rural	19.3	16.7	14.8	16.8	21.4	
Education (%)						<0.001
≤Middle school graduate	38.8	19.5	16.4	17.5	22.2	
High school graduate	32.4	37.6	36.6	35.6	35.9	
≥College graduate	28.8	42.9	47.0	46.9	41.9	
Nutrient intake						
Calorie intake (kcal/day)	1923.9 ± 853.7	2051.5 ± 845.1	2015.2 ± 788.8	2044.1 ± 799.7	1931.6 ± 787.4	<0.001
Calorie intake from carbohydrate (%)	64.7 ± 14.3	62.4 ± 13.4	62.5 ± 13.1	62.1 ± 13.3	62.2 ± 14.0	<0.001
Calorie intake from protein (%)	13.5 ± 4.1	14.1 ± 3.9	14.3 ± 4.2	14.3 ± 4.2	14.4 ± 4.1	<0.001
Calorie intake from fat (%)	17.7 ± 9.2	19.8 ± 8.9	20.4 ± 8.8	20.6 ± 9.2	20.7 ± 9.7	<0.001

^a^ Data are expressed as the percentage or mean ± standard deviation; ^b^ The *p*-value was obtained from a Chi-square test for categorical variables and one-way analysis of variance (ANOVA) for continuous variables.

**Table 2:** Adjusted odd ratios and 95% confidence intervals ^a^ for abdominal obesity according to sleep duration with the effect of fat intake.

	Sleep Duration (Hours/Day)						*p*-Trend ^a^
	*n*	≤5	6	7	8	≥9	
Aggregated Logit							
Model 1	13,686	Ref	0.88 (0.76–1.03)	0.72 ^‡^ (0.62–0.84)	0.74 ^‡^ (0.64–0.86)	0.65 ^‡^ (0.54–0.77)	<0.001
Model 2	13,686	Ref	0.88 (0.75–1.02)	0.72 ^‡^ (0.62–0.84)	0.74 ^‡^ (0.64–0.86)	0.65 ^‡^ (0.54–0.78)	<0.001
Model 3	13,686	Ref	0.88 (0.75–1.02)	0.72 ^‡^ (0.62–0.84)	0.74 ^‡^ (0.64–0.86)	0.65 ^‡^ (0.54–0.78)	<0.001
Stratified over proportion of calorie intake from fat ^b^							
Quartile 1 (9.8%)	3422	Ref	1.05 (0.81–1.37)	0.90 (0.70–1.17)	0.86 (0.66–1.13)	0.68 ^§^ (0.49–0.94)	0.007
Quartile 2 (16.4%)	3421	Ref	0.74 ^§^ (0.55–1.00)	0.53 ^‡^ (0.40–0.72)	0.68 ^§^ (0.51–0.92)	0.50 ^‡^ (0.34–0.73)	0.001
Quartile 3 (22.4%)	3422	Ref	0.78 (0.56–1.09)	0.74 (0.54–1.02)	0.59 ^†^ (0.42–0.82)	0.59 ^†^ (0.40–0.87)	0.001
Quartile 4 (30.8%)	3421	Ref	0.89 (0.63–1.27)	0.69 ^§^ (0.49–0.98)	0.82 (0.58–1.16)	0.84 (0.57–1.24)	0.406

^a^ Data and *p*-trend were obtained from a multivariable logistic regression model. Model 1 included sex, age, smoking, drinking, physical activity, family history of chronic disease, household income, living area and education; model 2 included the model 1 variables, in addition to calorie intake and the proportion of calorie intake from carbohydrates; model 3 included the model 2 variables in addition to the proportion of calorie intake from fat. *p*-Value ^§^ < 0.05, ^†^ < 0.01, ^‡^ < 0.001; ^b^ Multivariable logistic regression analyses on the stratified over proportion of calorie intake from fat was based on model 2.

**Table 3:** Adjusted odd ratios and 95% confidence intervals ^a^ for abdominal obesity according to sleep duration with the effect of stress.

	Sleep Duration (Hours/Day)						*p*-Trend ^a^
	*n*	≤5	6	7	8	≥9	
Aggregated Logit							
Model 1	13,686	Ref	0.88 (0.76–1.03)	0.72 ^‡^ (0.62–0.84)	0.74 ^‡^ (0.64–0.86)	0.65 ^‡^ (0.54–0.77)	<0.001
Model 2	13,686	Ref	0.88 (0.75–1.02)	0.72 ^‡^ (0.62–0.84)	0.74 ^‡^ (0.64–0.86)	0.65 ^‡^ (0.54–0.78)	<0.001
Model 3	13,686	Ref	0.90 (0.77–1.04)	0.74 ^‡^ (0.64–0.86)	0.76 ^‡^ (0.65–0.88)	0.66 ^‡^ (0.55–0.79)	<0.001
Stratified over self-reported stress level ^b^							
Rarely	1933	Ref	1.15 (0.77–1.71)	0.72 (0.48–1.07)	0.86 (0.58–1.28)	0.77 (0.49–1.20)	0.063
Slightly	8108	Ref	0.86 (0.70–1.07)	0.73 ^†^ (0.60–0.90)	0.70 ^†^ (0.57–0.86)	0.65 ^†^ (0.50–0.83)	<0.001
Moderately	3048	Ref	0.81 (0.60–1.10)	0.65 ^†^ (0.48–0.88)	0.76 (0.56–1.04)	0.51 ^‡^ (0.35–0.74)	0.002
Highly	597	Ref	0.71 (0.39–1.30)	1.09 (0.62–1.91)	0.98 (0.54–1.77)	1.04 (0.54–2.00)	0.606

^a^ Data and *p*-trend were obtained from a multivariable logistic regression model. Model 1 included sex, age, smoking, drinking, physical activity, family history of chronic disease, household income, living area and education; model 2 included the model 1 variables, in addition to calorie intake and the proportion of calorie intake from carbohydrates; model 3 included the model 2 variables, in addition to the self-reported stress level. *p*-Value ^†^ < 0.01, ^‡^ < 0.001; ^b^ Multivariable logistic regression analyses on the stratified over self-reported stress level was based on model 2.

## References

[1] Tchernof A., Després J.P. (2013). Pathophysiology of human visceral obesity: An update. Physiol. Rev..

[2] Lee C.M.Y., Huxley R.R., Wildman R.P., Woodward M. (2008). Indices of abdominal obesity are better discriminators of cardiovascular risk factors than BMI: A meta-analysis. J. Clin. Epidemiol..

[3] Janssen I., Katzmarzyk P.T., Ross R. (2004). Waist circumference and not body mass index explains obesity-related health risk. Am. J. Clin. Nutr..

[4] Ardern C.I., Katzmarzyk P.T., Janssen I., Ross R. (2003). Discrimination of health risk by combined body mass index and waist circumference. Obes. Res..

[5] National Sleep Foundation National Sleep Foundation Recommends New Sleep Times. https://www.sleepfoundation.org/press-release/national-sleep-foundation-recommends-new-sleep-times.

[6] OECD Average Minutes Per Day Spent Sleeping in OECD Countries by Gender. https://www.statista.com/statistics/521957/time-spent-sleeping-countries.

[7] Wu Y., Zhai L., Zhang D. (2014). Sleep duration and obesity among adults: A meta-analysis of prospective studies. Sleep Med..

[8] Sperry S.D., Scully I.D., Gramzow R.H., Jorgensen R.S. (2015). Sleep duration and waist circumference in adults: A meta-analysis. Sleep.

[9] Cappuccio F.P., Cooper D., D’Elia L., Strazzullo P., Miller M.A. (2011). Sleep duration predicts cardiovascular outcomes: A systematic review and meta-analysis of prospective studies. Eur. Heart J..

[10] Liu T.Z., Xu C., Rota M., Cai H., Zhang C., Shi M.J., Yuan R.X., Weng H., Meng X.Y., Kwong J.S. (2017). Sleep duration and risk of all-cause mortality: A flexible, non-linear, meta-regression of 40 prospective cohort studies. Sleep Med. Rev..

[11] Fatima Y., Doi S.A., Mamun A.A. (2015). Longitudinal impact of sleep on overweight and obesity in children and adolescents: A systematic review and bias-adjusted meta-analysis. Obes. Rev..

[12] Itani O., Jike M., Watanabe N., Kaneita Y. (2017). Short sleep duration and health outcomes: A systematic review, meta-analysis, and meta-regression. Sleep Med..

[13] Irwin M.R., Olmstead R., Carroll J.E. (2016). Sleep disturbance, sleep duration, and inflammation: A systematic review and meta-analysis of cohort studies and experimental sleep deprivation. Biol. Psychiatry.

[14] Lin C.L., Lin C.P., Chen S.W., Wu H.C., Tsai Y.H. (2018). The association between sleep duration and overweight or obesity in Taiwanese adults: A cross-sectional study. Obes. Res. Clin. Pract..

[15] Sasaki N., Fujiwara S., Yamashita H., Ozono R., Monzen Y., Teramen K., Kihara Y. (2018). Association between obesity and self-reported sleep duration variability, sleep timing, and age in the Japanese population. Obes. Res. Clin. Pract..

[16] Chaput J.P., Després J.P., Bouchard C., Tremblay A. (2007). Short sleep duration is associated with reduced leptin levels and increased adiposity: Results from the québec family study. Obesity.

[17] Spiegel K., Tasali E., Penev P., Cauter E.V. (2004). Brief communication: Sleep curtailment in healthy young men is associated with decreased leptin levels, elevated ghrelin levels, and increased hunger and appetite. Ann. Intern. Med..

[18] Taheri S., Lin L., Austin D., Young T., Mignot E. (2004). Short sleep duration is associated with reduced leptin, elevated ghrelin, and increased body mass index. PLoS Med..

[19] Balbo M., Leproult R., Van Cauter E. (2010). Impact of sleep and its disturbances on hypothalamo-pituitary-adrenal axis activity. Int. J. Endocrinol..

[20] George S.A., Khan S., Briggs H., Abelson J.L. (2010). CRH-stimulated cortisol release and food intake in healthy, non-obese adults. Psychoneuroendocrinology.

[21] St-Onge M.P., Roberts A.L., Chen J., Kelleman M., O’Keeffe M., Choudhury A.R., Jones P.J.H. (2011). Short sleep duration increases energy intakes but does not change energy expenditure in normal-weight individuals. Am. J. Clin. Nutr..

[22] Brondel L., Romer M.A., Nougues P.M., Touyarou P., Davenne D. (2010). Acute partial sleep deprivation increases food intake in healthy men. Am. J. Clin. Nutr..

[23] Nedeltcheva A.V., Kilkus J.M., Imperial J., Kasza K., Schoeller D.A., Penev P.D. (2009). Sleep curtailment is accompanied by increased intake of calories from snacks. Am. J. Clin. Nutr..

[24] Shi Z., McEvoy M., Luu J., Attia J. (2008). Dietary fat and sleep duration in Chinese men and women. Int. J. Obes..

[25] Mozaffarian D., Hao T., Rimm E.B., Willett W.C., Hu F.B. (2011). Changes in diet and lifestyle and long-term weight gain in women and men. N. Engl. J. Med..

[26] Kim S., DeRoo L.A., Sandler D.P. (2011). Eating patterns and nutritional characteristics associated with sleep duration. Public Health Nutr..

[27] Kant A.K., Graubard B.I. (2014). Association of self-reported sleep duration with eating behaviors of American adults: NHANES 2005–2010. Am. J. Clin. Nutr..

[28] Kohsaka A., Laposky A.D., Ramsey K.M., Estrada C., Joshu C., Kobayashi Y., Turek F.W., Bass J. (2007). High-fat diet disrupts behavioral and molecular circadian rhythms in mice. Cell Metabol..

[29] Eckel-Mahan K.L., Patel V.R., de Mateo S., Orozco-Solis R., Ceglia N.J., Sahar S., Dilag-Penilla S.A., Dyar K.A., Baldi P., Sassone-Corsi P. (2013). Reprogramming of the circadian clock by nutritional challenge. Cell.

[30] Patel S.R., Malhotra A., White D.P., Gottlieb D.J., Hu F.B. (2006). Association between reduced sleep and weight gain in women. Am. J. Epidemiol..

[31] Nishiura C., Noguchi J., Hashimoto H. (2010). Dietary patterns only partially explain the effect of short sleep duration on the incidence of obesity. Sleep.

[32] Minkel J., Moreta M., Muto J., Htaik O., Jones C., Basner M., Dinges D. (2014). Sleep deprivation potentiates HPA axis stress reactivity in healthy adults. Health Psychol..

[33] Vgontzas A.N., Lin H.M., Papaliaga M., Calhoun S., Vela-Bueno A., Chrousos G.P., Bixler E.O. (2008). Short sleep duration and obesity: The role of emotional stress and sleep disturbances. Int. J. Obes..

[34] Knudsen H.K., Ducharme L.J., Roman P.M. (2007). Job stress and poor sleep quality: Data from an American sample of full-time workers. Soc. Sci. Med..

[35] Sgoifo A., Buwalda B., Roos M., Costoli T., Merati G., Meerlo P. (2006). Effects of sleep deprivation on cardiac autonomic and pituitary-adrenocortical stress reactivity in rats. Psychoneuroendocrinology.

[36] Dallman M.F., Pecoraro N., Akana S.F., La Fleur S.E., Gomez F., Houshyar H., Bell M.E., Bhatnagar S., Laugero K.D., Manalo S. (2003). Chronic stress and obesity: A new view of “comfort food”. Proc. Natl Acad. Sci. USA.

[37] Kuo L.E., Czarnecka M., Kitlinska J.B., Tilan J.U., Kvetansk R., Zukowska Z. (2008). Chronic stress, combined with a high-fat/high-sugar diet, shifts sympathetic signaling toward neuropeptide Y and leads to obesity and the metabolic syndrome. Ann. N. Y. Acad. Sci..

[38] Lee S.Y., Park H.S., Kim D.J., Han J.H., Kim S.M., Cho G.J., Kim D.Y., Kwon H.S., Kim S.R., Lee C.B. (2007). Appropriate waist circumference cutoff points for central obesity in Korean adults. Diabets Res. Clin. Pract..

[39] Corraini P., Olsen M., Pedersen L., Dekkers O., Vandenbroucke J. (2017). Effect modification, interaction and mediation: An overview of theoretical insights for clinical investigators. Clin. Epidemiol..

[40] Jager K.J., Zoccali C., MacLeod A., Dekker F.W. (2008). Confounding: What it is and how to deal with it. Kidney Int..

[41] Greene (2012). Econometric Analysis.

[42] MacKinnon D.P., Krull J.L., Lockwood C.M. (2000). Equivalence of the mediation, confounding and suppression effect. Prev. Sci..

